# Can Smartphone-Derived Step Data Predict Laboratory-Induced Real-Life Like Fall-Risk in Community- Dwelling Older Adults?

**DOI:** 10.3389/fspor.2020.00073

**Published:** 2020-07-10

**Authors:** Yiru Wang, Rachana Gangwani, Lakshmi Kannan, Alison Schenone, Edward Wang, Tanvi Bhatt

**Affiliations:** ^1^Department of Physical Therapy, College of Applied Health Sciences, University of Illinois at Chicago, Chicago, IL, United States; ^2^MS Program in Rehabilitation Sciences, Department of Physical Therapy, College of Applied Health Sciences, University of Illinois at Chicago, Chicago, IL, United States; ^3^Ph.D. Program in Rehabilitation Sciences, Department of Physical Therapy, College of Applied Health Sciences, University of Illinois at Chicago, Chicago, IL, United States

**Keywords:** fall prediction, steps data, smartphone technology, falls, older adults

## Abstract

**Background:** As age progresses, decline in physical function predisposes older adults to high fall-risk, especially on exposure to environmental perturbations such as slips and trips. However, there is limited evidence of association between daily community ambulation, an easily modifiable factor of physical activity (PA), and fall-risk. Smartphones, equipped with accelerometers, can quantify, and display daily ambulation-related PA simplistically in terms of number of steps. If any association between daily steps and fall-risks is established, smartphones due to its convenience and prevalence could provide health professionals with a meaningful outcome measure, in addition to existing clinical measurements, to identify older adults at high fall-risk.

**Objective:** This study aimed to explore whether smartphone-derived step data during older adults' community ambulation alone or together with commonly used clinical fall-risk measurements could predict falls following laboratory-induced real-life like slips and trips. Relationship between step data and PA questionnaire and clinical fall-risk assessments were examined as well.

**Methods:** Forty-nine community-dwelling older adults (age 60–90 years) completed Berg Balance Scale (BBS), Activities-specific Balance Confidence scale (ABC), Timed Up-and-Go (TUG), and Physical Activity Scale for the Elderly (PASE). One-week and 1-month smartphone steps data were retrieved. Participants' 1-year fall history was noted. All participants' fall outcomes to laboratory-induced slip-and-trip perturbations were recorded. Logistic regression was performed to identify a model that best predicts laboratory falls. Pearson correlations examined relationships between study variables.

**Results:** A model including age, TUG, and fall history significantly predicted laboratory falls with a sensitivity of 94.3%, specificity of 58.3%, and an overall accuracy of 85.1%. Neither 1-week nor 1-month steps data could predict laboratory falls. One-month steps data significantly positively correlated with BBS (*r* = 0.386, *p* = 0.006) and ABC (*r* = 0.369, *p* = 0.012), and negatively correlated with fall history (*r*_p_ = −0.293, *p* = 0.041).

**Conclusion:** Older participants with fall history and higher TUG scores were more likely to fall in the laboratory. No association between smartphone steps data and laboratory fall-risk was established in our study population of healthy community-dwelling older adults which calls for further studies on varied populations. Although modest, results do reveal a relationship between steps data and functional balance deficits and fear of falls.

## Introduction

Falls are a common and a serious problem in older adults aged 60 years and above (Rubenstein, 2006; Carpenter et al., 2019). Even the healthiest community-dwelling older adults are not immune to falls, especially on exposure to external environmental perturbations such as slips and trips which accounts for 60% of outdoor falls among community-dwelling older adults aged 70 years and above (Berg et al., 1997; Luukinen et al., 2000; Crenshaw et al., 2017). Such falls occur due to age-related physiological changes resulting in balance dysfunction, reduced muscle strength, and impaired gait pattern, predisposing older adults to high fall-risk (Ambrose et al., 2013; Zhao et al., 2018). Additionally, with progressing age, older adults experience a decline in physical function and activities of daily living which further increases fall-risk (Smee et al., 2012; Welmer et al., 2017). Falls result in several deleterious physical consequences such as fractures and soft tissue injuries but also lead to fear of falling, thereby resulting in further self-imposed restriction of physical activity (PA) (Pereira et al., 2014; Young and Mark Williams, 2015).

Apart from aging, a fall-risk factor which is non-modifiable, PA which can mitigate age-related declines in muscle strength, balance, and agility is considered a modifiable risk-factor. Due to its adaptability, PA could be systematically monitored and enhanced in the community-living geriatric population to reduce fall-risk. Although sparse, evidence using the self-reported questionnaire, Physical Activity Scale for the Elderly (PASE), demonstrated that fallers had lower PASE scores (less PA) compared to non-fallers and that the low scores were associated with high fall-risk and fear of falling (Roig et al., 2011; Oliveira et al., 2017). Such a questionnaire-based assessment can serve as an inexpensive tool to assess PA among community-dwelling older adults over a period of 7 days (Washburn et al., 1993; Logan et al., 2013; Duray and Genc, 2017). However, it has several limitations. Firstly, self-reported techniques show recall bias, especially when used by older adults with possibly declining memory. Additionally, the need to give socially desirable answers can affect the accuracy of results (Perell et al., 2001). Finally, studies suggest that PASE might have a floor effect because several activities listed in PASE, such as outdoor gardening, yard work, painting, and wall papering, might not be commonly performed activities by older adults (Sallis and Saelens, 2000).

Contrary to self-reported measures, wearable sensor technology comprising of research-based (ActivPal and ActiGraph) and commercially available (FitBit and Apple watch) motion sensors automatically track and store PA and thus effectively combat the issue of recall-bias. Such accelerometer-based wearable sensors are able to identify PA patterns (frequency, duration, and intensity) under both controlled laboratory conditions and uncontrolled, realistic conditions of daily living (Plasqui and Westerterp, 2007; Gomersall et al., 2016; Rosenberger et al., 2016). Additionally, commercially available wearable sensor technology records and stores PA simply in terms of number of steps for several months and years. Thus, commercially available wearable sensors have gained popularity for PA monitoring in both young and older adult populations. The advantage of such technology is that step count can be easily interpreted by older adults themselves or by clinicians and used for comparative analysis by researchers. Among various PA parameters (distance covered, number of steps, and energy expenditure), step data has become the hallmark measure of PA monitoring (Tudor-Locke et al., 2005). One probable reason that steps are being used to represent PA is because walking is one of the most commonly reported forms of activity performed even among sedentary older adults (Paillard et al., 2004). In addition, other than its significant health benefits, walking has become a focus for public health interventions because of its feasibility and acceptability (Li et al., 2005).

Although commercially available accelerometer-based wearable sensors have several advantages, they have demonstrated reduced long-term compliance in older adults (compared to the younger population) due to the need of carrying an extra device and sometimes the cost associated with it (Marschollek et al., 2011; Ferrari et al., 2012). Thus, with the advancement in smartphone technology, the latter have replaced or supplemented wearable sensors for PA monitoring. Smartphones equipped with tri-axial accelerometers can be used in conjunction with inbuilt or freely available smartphone applications for measuring and recording step data. Studies indicate that smartphone applications similar to wearable sensor technology can deliver step data in a user-friendly interface (Higgins, 2016; Lu et al., 2017). For example, Harries et al. reported that participants had greater adherence to using the smartphone applications than wearing their wearable devices because running the smartphone application was quite simple and did not require much effort (Harries et al., 2016). In addition to their easy implementation, studies demonstrated that the accuracy of step data collected using smartphones was just as high as accelerometer-based sensors (Higgins, 2016; Lu et al., 2017).

Although the significance of PA as a modifiable fall-risk factor is established (Chan et al., 2007; Pereira et al., 2008), to the authors' knowledge there is limited evidence of association between daily life ambulation and fall-risk. Studies that demonstrated the association of daily-life ambulation and fall-risk (Rispens et al., 2015; van Schooten et al., 2015), utilized wearable-sensor technology and determined various daily-life ambulatory gait parameters that can predict fall-risk in older adults. However, they did not take number of steps into consideration. Limited studies have considered using number of steps as a parameter to identify fallers. Brodie et al. (2015) found that shorter ambulatory periods with fewer steps recorded using a wearable sensor can identify older adult fallers. However, another study by Weiss et al. (2013) did not find any significant difference between fallers and non-fallers based on the number of steps. Secondly, most fall-risk prediction studies involve retrospective or prospective data of real-life falls collected subjectively via a fall diary. Such a method can introduce a recall bias on the number and type of falls (i.e., the cause of falls). Usually, a large sample of participants are needed to be monitored consistently over a long period of time to collect sufficient fall events. With recent advances in technology, it is possible to reproduce slip and trip-like falls that closely resemble those encountered in daily life (albeit in safe laboratory conditions) to determine the prognostic capacity of various fall-risk measures (Bhatt et al., 2011). Thus, such experimental method allows for an immediate and quantifiable investigation of participants' susceptibility to certain types (i.e., slip and trip) of falls over a short test session.

In summary, free-living steps data, an easily accessible aspect of PA, collected by smartphone could largely increase the feasibility of studies on PA in the geriatric population under realistic community-living conditions. With the ability to reproduce real-life like falls in a laboratory environment, we could explore the association between daily steps data and fall-risk without the need to conduct a longitudinal study with community-based monitoring. If any association between daily steps taken and fall-risk is established, smartphone-based PA monitoring could provide health professionals with a meaningful outcome measure, in addition to existing clinical measurements, to better identify older adults at high fall-risk.

Thus, this study examined whether smartphone-derived steps data either as a single factor or along with other commonly used clinical fall-risk measures could predict laboratory-induced slip or trip related fall-risk in older adults. We also examined if the steps data would correlate with the questionnaire-based PA assessment, the PASE, and with other commonly used clinical fall-risk measures. Additionally, the study included a sub-analysis to determine the prediction capacity of both 1-week step data vs. 1-month step data to better understand the time dependency, if any on the predictive and associative relationships of step data.

## Method

### Participants

Community-dwelling ambulatory healthy older adults were recruited within a 50-mile radius from the laboratory in the city and the neighboring suburbs of the Greater Chicago Area. The study participants were recruited through advertisements via study flyers distributed at different senior centers, community exercise centers, and independent senior living facilities. Participants were included in the study if they were at least 60 years old, weighed <250 pounds, received a cognitive score of >25 on the Folstein Mini Mental Status Exam (MMSE), possessed a smartphone with the “Google Fit” application for Android phones or “Health” application for iPhones for steps data collection, and if they had installed and enabled their respective application for at least 1 month prior to screening. Exclusion criteria included participants with acute (<6 months) musculoskeletal conditions such as back pain or fracture or having a surgical history 6 months prior to the laboratory perturbation test. Seventy-six participants agreed to participate in this pilot study and were included in the initial screening. Participants were excluded if they did not pass the initial screening test (*n* = 9), did not have entire 1 month smartphone data (*n* = 8) or if the smartphone steps data was not recorded even if the participants mentioned that they carried their phones (*n* = 5) or had incomplete laboratory data due to missing markers (*n* = 5). Ultimately, 49 participants were included in the final analysis (Table 1). All participants provided written informed consent and this study was approved by the Institutional Review Board.

**Table 1:** Sample demographics and baseline clinical measurements with the mean and standard deviations.

**Variables**	**Fallers (*N* = 36)**	**Non-fallers (*N* = 13)**
	**Mean (*SD*)**	**Mean (*SD*)**
Age (y)	71.72 (5.56)	66.92 (5.15)
Weight (lbs)	160.73 (30.41)	152.84 (30.77)
Height (m)	1.64 (0.81)	1.71 (0.85)
TUG (s)	8.10 (1.23)	7.64 (1.21)
BBS (out of 56)	53.69 (2.05)	53.46 (2.36)
Fall history (%)	47%	38%
ABC (%)	87.80 (11.47)	85.16 (12.55)
PASE	129.57 (62.60)	159.54 (70.29)
MMSE	29.5 (0.77)	28.92 (1.65)
1-week steps	30534 (17637.5)	34286 (18544.5)
1-month steps	131528 (79996.9)	151004 (79328.5)
- <1,00,000 steps	62257.5 (23476.2)	62139 (27319)
−1,00,000–2,00,000	139899.6 (33210)	145497 (26911)
- >2,00,000 steps	270834 (55744.2)	256759.7 (39458)

### Study Design, Protocol, and Outcome Variables

**Figure 1:**
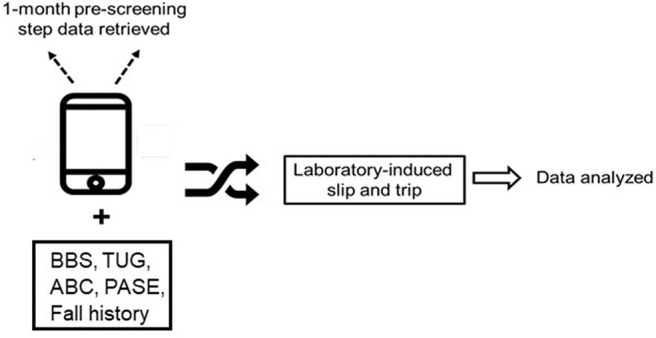
This figure demonstrates the study protocol. Participants were subjected to various fall-risk assessment measures such as Berg Balance Scale (BBS), Activities specific balance confidence scale (ABC), Physical activity scale for elderly (PASE), and previous 1-year fall history. One-month smartphone steps data was also retrieved. Participants were subjected to various fall-risk assessment measures such as the Berg Balance Scale (BBS), Activities specific Balance Confidence scale (ABC), Timed Up and Go (TUG), Physical Activity Scale for Elderly (PASE) and previous 1-year fall history. One week and one-month smartphone steps data was also retrieved.

On the initial screening day, all participants underwent various clinical measures to assess their balance [Berg Balance Scale (BBS)], balance confidence [Activities-specific Balance Confidence scale (ABC)], functional mobility [Timed Up-and-Go test (TUG)], and PA [Physical Activity Scale for the Elderly (PASE)]. The BBS assesses balance during functional tasks, and the scores range from 0 to 56 with higher scores indicating better balance and lower fall-risk (Whitney et al., 1998; Steffen et al., 2002). The ABC scale assesses balance confidence across 16 activities, and the scores range from 0 to 100% with higher percentages indicating a higher level of balance confidence (Powell and Myers, 1995; Myers et al., 1998). The TUG score represents the time taken to stand up from a chair, walk a distance of 3-m, turn around, and sit back on the chair. Higher scores represent greater time taken to complete the test, indicating poor functional mobility, and high fall-risk (Shumway-Cook et al., 2000; Bohannon, 2006). The PASE scores were calculated from weights and frequency values for each of the 12 types of activities, and a higher score indicates greater PA (Washburn et al., 1993). Self-recalled fall history for the past 1 year was also obtained (Figure 1).

**Figure 2:**
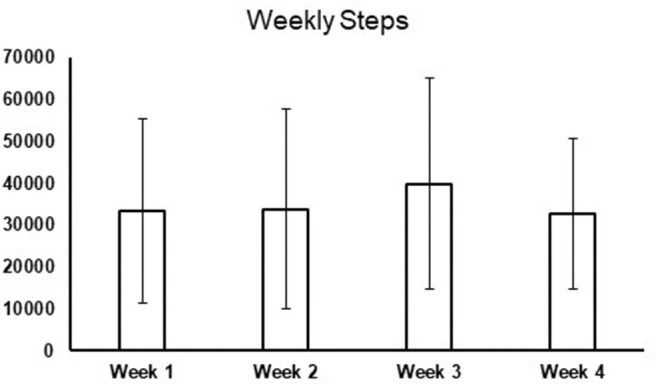
This figure demonstrates the total number of steps walked each week for 4 weeks (1 month) to show that 1-month steps data provided consistent information regarding the participant's physical activity behavior throughout 4 weeks.

In addition, on the day of the initial screening the total steps for the past 1-week and 1-month were retrieved and summed from participants' smartphones. Figure 2 presents the number of steps walked each week for 4 weeks to indicate that consistent data was collected thereby depicting their consistent PA behavior. Based on the sum of 1-month step data collected for each participant, we classified the data in 3 sub-categories (<1,00,000 steps, 1,00,000–2,00,000 steps, and more than 2,00,000 steps). A qualitative questionnaire including four questions were asked (1) number of hours the participant carries his or her phone, (2) the time of day when the participant is most active, (3) whether the participant carries his or her phone all the time they were active, and (4) whether he or she owns a wearable fitness tracker such as FitBit or Apple watch. All participants were then scheduled to receive the laboratory slip and trip perturbations within 2 weeks of the initial screening date (Figure 1).

### Laboratory Fall Test

**Figure 3:**
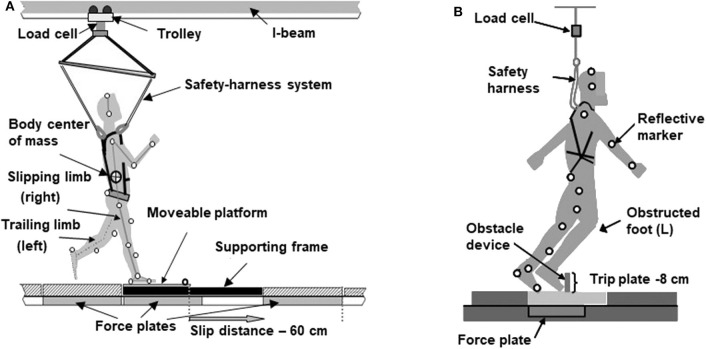
**(A)** Schematic diagram that indicates the experimental slip set-up demonstrating the participant in a safety harness attached to the load cell and reflective markers attached to the anatomical landmarks. As the right heel of the participant land on the moveable platform, it is unlocked to slide up to a distance of 60 cm. **(B)** The experimental trip set-up demonstrating the trip plate in its upright position as an obstacle to induce a trip.

During the laboratory session, participants were assigned to receive a novel slip and trip perturbation in random order. Participants first had to walk 25–35 unperturbed baseline trials to become familiar with the laboratory walking environment before receiving their novel slip or trip perturbation. Each participant received a single slip and a single trip given in a random order. Thus, the participants experienced a total of two perturbations while undergoing the laboratory test. They were informed “a slip or trip may or may not occur during walking.” The starting position was adjusted during baseline walking to ensure the upcoming slip or trip trials were induced properly. The slip was induced by a pair of low-friction movable platforms imbedded in a 7-meter walkway. These platforms were mounted on top of low-friction aluminum tracks resting on four force plates (AMTI, Newton, MA). The unannounced release of the platform occurred at heel strike of the perturbed (right) limb, and, following the platform's release, it was free to slide a distance of up to 60 cm (Wang et al., 2019a). Such slip distance has been reported to be enough to induce a fall in older adults (Figure 3A). The laboratory trip was induced on the left side by a hinged metal plate imbedded in the same walkway. During regular walking, the plate was locked in a flat position by a pair of electromagnets. For the trip trial, the electromagnets that kept the metal plate in a flat position were powered off to unlock the plate and the springs returned the plate to an upright position to induce a trip when the vertical ground reaction force (GRF) under the unperturbed (right) limb exceeded 80% of the participant's body weight after right heel strike (Figure 3B; Wang et al., 2012). All participants were protected by a safety harness connected through a load cell (Transcell Technology Inc., Buffalo Grove, IL) to a low-friction trolley-and-beam system mounted to the ceiling along the walking path. A fall was determined if the load cell detected more than 30% of the participant's body weight after the slip or trip (Yang and Pai, 2011). Additionally, the perturbation outcome was determined to be a fall based on the video recording if the participant was visually observed to have fallen after the novel slip or trip.

### Statistical Analysis

Data was summarized using descriptive statistics (means and standard deviations) for all variables including demographics, that is, age, height and weight, fall-risk measurements such as BBS, TUG, ABC, PASE as well as previous 1-year fall history and 1-week and 1-month smartphone steps. In addition, means and standard deviations were also calculated for each sub-category based on 1-month data (<1,00,000 steps, 1,00,000–2,00,000 steps, and more than 2,00,000 steps). Paired *t*-tests were performed between faller and non-faller groups for overall 1-week and 1-month step data as well as its subcategories to determine whether there is a significant difference in step data between fallers and non-fallers (Lee et al., 2019). Multiple univariate logistic regression analyses were performed to individually identify variables that could best predict laboratory fall-risk (induced by a slip and trip trial) in older adults. Laboratory fall outcome was treated as a binominal variable with the outcome for participants who fell in either one or both of the two perturbations being denoted as 1 or else with a 0. Hours of phone carriage was also inputted into the logistic regression as a covariate. Based on the univariate logistic regression results, variables with a significance of ≤0.1 were included in the multivariate logistic regression analysis (Bursac et al., 2008; Sperandei, 2014). A multivariate logistic regression analysis using a backward stepwise method was performed to generate a model with variables that could best predict laboratory-induced slip or trip induced falls in older adults. A receiver operating characteristic (ROC) curve was used to determine the cut-off scores (a score with the highest sensitivity and specificity) of significant variables in the univariate logistic regression and to determine the area under the curve (AUC) for the overall model predicted by the multivariate logistic regression. Pearson correlations were conducted to examine the relationships between participants' demographics, total 1-week and 1-month smartphone collected steps, fall-risk measurements, and PASE. Point biserial correlation was applied to examine relationships between steps data and the dichotomous fall histories.

## Results

Based on the results of the qualitative questionnaire, 12 older adults used Android phones whereas 37 used an iPhone. On average, participants carried their phones 9 h (9.06 ± 5.6 h) per day. Of all the participants, 38 reported they were active most of the time they carried their phones, 6 reported they were active even during the time they did not carry their phones, and 5 were unable to recall or answer the question. Only 11 participants owned a wearable device such as a Fitbit.

Out of the 49 participants, 35 participants fell on at least one perturbation during the laboratory fall test. Thirty-two participants fell only on the slip perturbation, 18 participants fell only on the trip perturbation, and 14 participants fell on both slip and trip perturbations. Table 1 indicates the means of demographic data and outcome measures of fallers and non-fallers groups. Based on the results of the paired *t*-test there was no significant difference in overall 1-week and 1-month step data or its sub-categories between fallers and non-fallers (*p* > 0.05). Based on the univariate regression analyses, age, and TUG were the only significant variables with the significance value set at *p* = 0.05 (Table 2) and fall history having a near significant value of *p* = 0.056. Based on the results of the ROC curve, we established that the variable age with a cut-off score of 69.5 had a sensitivity of 63.9% and specificity of 61.5%, indicating that older adults above 69.5 years had a greater fall-risk. Similarly, for the variable TUG, the cut off score of 7.49 had a sensitivity of 80% and specificity of 58.3%, indicating that older adults who took longer than 7.49 s to complete the TUG test were at greater fall-risk.

**Table 2:** Variables and their significance (*p*-value) and *R* square value based on univariate logistic regression results.

**Variable**	***p*-value**	***R* square value**
Age	0.011	0.147
Weight	0.592	0.006
Fall History	0.056	0.075
MMSE	0.177	0.036
ABC	0.313	0.022
BBS	0.820	0.001
TUG	0.043	0.100
PASE	0.320	0.020
1-week steps	0.863	0.003
1-month steps	0.198	0.040

**Figure 4:**
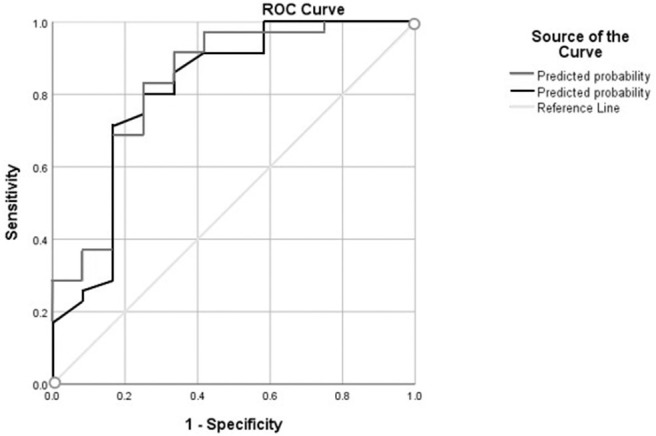
This figure demonstrates the area under the curve (AUC) for model 1 (area under the black line) comprising of variables age and fall history and model 2 (area under the gray line) comprising of variable age, fall history, and Timed Up and Go (TUG) found using the Receiver Operating Curve (ROC).

Furthermore, variables with a significance of ≤0.1 for the univariate logistic regression analysis were included in the multivariate logistic regression analysis using the backward stepwise method (Table 2). The multivariate regression analysis revealed an overall model (Model 1) including age and fall history with an overall accuracy of 83% to predict laboratory-induced falls with a sensitivity of 97.1% and specificity of 41.7% (*p* = 0.002). The area under the curve (AUC) for the model was 0.807. A model before the final model included TUG in addition to variables age and fall history (Model 2). Addition of TUG in the final model (Model 2) improved the overall accuracy to 85.1% with a sensitivity of 94.3% and a better specificity of 58.3% (*p* = 0.002). The AUC of this model increased to 0.831 (Table 3). Figure 4 indicates the AUC for both the models.

**Table 3:** Overall model predicted based on multivariate logistic regression results along with the sensitivity, specificity, overall accuracy, and the area under the curve (AUC) found using the Receiver Operating Curve (ROC).

**Model**	**Variable**	**Significance**	**Sensitivity**	**Specificity**	**Overall accuracy**	**Overall significance**	**AUC**
1	Age	0.006	97.1	41.7	83.0	0.002	0.807
	Fall history	0.0065					
2	Age	0.012	94.3	58.3	85.1	0.002	0.831
	TUG	0.169
	Fall history	0.122					

Thus, participants who were older, had a higher TUG score (took longer to complete the test), and had a history of falls were more likely to fall during the laboratory-induced perturbations. The best logistic regression model which predicted immediate laboratory fall is represented as Predicted Logic of (Laboratory fall) = 18.175 + (0.207)^*^Age + (0.525)^*^TUG + (1.324)^*^History of fall. Based on this, the odds of falling for a person who had a fall history in the previous year would be 3.75 times more than those with no falls. Similarly, for each 1 s increase in TUG score (slower), the odds of experiencing perturbation-induced falls would be 1.69 times higher. Further, for every 1-year increase in age, the odds of falling on laboratory perturbation would be 1.22 times higher.

Pearson correlations revealed a significant negative correlation between age and hours of phone carriage (*r* = −0.300, *p* = 0.046), indicating that the older participants carried their phones for less time than the younger ones. One-week steps data did not correlate with any clinical fall-risk measure and history of fall. However, 1-month steps data positively correlated with higher BBS (*r* = 0.386, *p* = 0.006) and ABC (*r* = 0.369, *p* = 0.012) scores and negatively correlated with previous 1-year fall history (*r*_pb_ = −0.293, *p* = 0.041). Additionally, hours of phone carriage positively correlated with steps data (*r* = 0.327, *p* = 0.028) and, hence, was inputted as a covariate in logistic regression. There was no significant correlation between steps data and PASE score.

## Discussion

This study explored the relationship between smartphone steps data and other commonly used clinical fall-risk measures and determined their ability to predict laboratory-induced slip or trip falls in healthy community-dwelling older adults. Univariate logistic regression analyses predicted age and TUG as individual significant predictors of laboratory falls with fall history having a near significance value. Multivariate logistic regression determined a model, which included age and fall history that best predicted laboratory-induced slip or trip fall-risk. Addition of TUG to the final model improved the overall prediction capacity of the model. Neither 1-week nor 1-month steps data could predict laboratory-induced fall-risk as a single predictor or in the multivariate model. A weak but significant positive correlation was noted between 1-month smartphone steps and BBS and ABC scores and a negative correlation was seen with previous 1-year fall history. No correlation was found between smartphone steps and PASE scores or other fall-risk measures.

Based on the multivariate logistic regression results, the final model predicted included age and fall history (Model 1) with a sensitivity of 97.1%, specificity of 47.1%, overall accuracy of 83%, and AUC of 0.807, indicating that older adults with a previous history of fall were predisposed to a greater fall-risk during laboratory-induced perturbations. Our results are consistent with the previous literature indicating that older adults with a fall history have difficulty maintaining postural control and thus are at a higher fall-risk (Horak et al., 1989; Ambrose et al., 2013). Similarly, the relationship between aging and falls is consistent with previous literature which indicated that fall rates increase with aging (Ageing Life Course family Community Health World Health Organization, 2008). For example, Pai et al. (2010) demonstrated reduced stability control in older adults compared to young adults, thereby predisposing them to a greater risk for falls during laboratory-induced slip perturbations. Furthermore, aging also has an effect on the recovery stepping response which is critical for establishing a new functional base of support following a perturbation thereby further increasing fall-risk (Tseng et al., 2009).

A model before the final model included TUG in addition to variables age and fall history (Model 2) with similar sensitivity of 94.3%, a better specificity of 58.3%, overall accuracy of 85.1%, and a higher AUC of 0.831. Additionally, TUG as a single factor had significant prediction for laboratory-induced falls, indicating that participants who took longer to finish the test had greater risk for laboratory-induced falls. Our results were similar to a previous study, wherein TUG was able to independently predict 60% of slip-induced falls (Bhatt et al., 2011), and these falls resulted in the center of mass (COM) moving behind the forwardly sliding base of support (BOS). Thus, a lower TUG score could indicate a superior ability of the participant to rapidly relocate the COM over the displaced BOS resulting in improved COM state stability against slip-induced balance loss (Pai and Iqbal, 1999). Conversely, participants would experience forward instability following a trip perturbation due to the forward shift of both COM velocity and displacement with respect to the BOS. Therefore, a faster walking speed could increase forward instability, resulting in a greater risk for trip-induced falls (Pavol et al., 2001; Wang et al., 2019b). The TUG test scores could thus have had opposing predictive effects for slip vs. trip perturbation. In spite of the possibility of such an opposing relationship between TUG scores and fall-type, TUG emerged as a significant fall-risk predictor probably due to the fact that there were greater slip falls (*n* = 32) than trip falls (*n* = 18). However, in spite of being a significant predictor in the univariate regression analyses, it was not included in the final multivariate logistic regression model comprising of age and fall history probably due to the opposite effect of walking speed on the recovery of slip and trip explained above (Model 1). Even though TUG was included in the second model, surprisingly the individual significance of the variables fall history and TUG was lost. This might possibly be because in Multivariate logistic regression analysis, the two variables are basically “competing” with each other for explaining laboratory falls.

Although smartphones are prevalent and can be readily used in the community, steps data collected were unable to accurately predict laboratory falls. Additionally, our results indicated no difference in total 1-week, 1 month, or sub-category step data between fallers and non-fallers. Thus, indicating that either there was no association between daily steps, a single aspect of PA, and fall-risk (given the steps collected by smartphones were accurate) or smartphone is an insufficient tool in collecting steps data under free-living condition. Our study survey indicated that only 22.44% (11/49) of participants in the current study had access to an extra wearable PA tracker (Fitbit). Thus, older adults majorly relied on smartphones for PA monitoring. However, a few study participants mentioned in the survey that they only carried their phones when going outside. Thus, we might have missed out on data when older adults were physically active but not carrying their phones, especially when they were walking inside their homes while performing daily routine activities. Moreover, participants were not given any instruction by the experimenter on the way they should carry their smartphone. Thus, the participants could have carried their smartphones in many different manners, and the phones could have recorded steps differently based on their position and orientation (Carter et al., 2018; Funk and Karabulut, 2018). A recent study also suggested that smartphone updates and different application versions might potentially change the outcomes of smartphone-based assessments (Brodie et al., 2018). It is also unclear whether the type of smartphone application matters, and if there was a difference between the “Health” and “Google Fit” applications since both were used in the study.

PA assessed using PASE was not a significant predictor of fall-risk. The PASE questionnaire might not accurately predict fall-risk in older adults as it only monitors PA over a span of 7 days, which might not be enough to provide an overall view of older adults' PA. As mentioned earlier, recall bias involved in such self-report technique might also limit the accuracy of PA measurement. Additionally, studies have demonstrated reporting bias for subjective questionnaires like PASE, such as individuals overestimating the time spent on strenuous activities or underestimating the time spent on activities that require less exertion (Bolszak et al., 2014). Also, such subjective questionnaires might lead to participants giving socially desirable answers. As PA is encouraged in older adults, participants might overestimate their PA to attain social approval. Such behavior would provide inaccurate data, thus further limiting PASE's sensitivity for fall prediction. Furthermore, PASE involves scoring an individual based on their frequency scores obtained for moderate to strenuous activities which may not be commonly performed by older adults. Thus, PA assessments should comprise of activities performed frequently by older adults to provide better fall-risk prediction. Measurement of these activities might also explain why PASE scores did not correlate with smartphone steps, as PASE considered the overall PA of older adults, including everyday household, recreational, and occupational activities, whereas smartphones only considered steps data.

BBS was not a significant predictor of falls in healthy older adults, which is consistent with the previous literature (Mancini and Horak, 2010; Bhatt et al., 2011). Previous studies suggest that individuals with a BBS cut-off score of 45 and above are high-functioning and at a lower risk of falls (Berg et al., 1992a, 1997). However, despite the average BBS score in our study sample being 53.57, a score much higher than the threshold suggesting a low risk of fall (Berg et al., 1992b), 36 participants out of 49 still fell during at least one laboratory-induced perturbation and 22 had previous fall histories, indicating the limited sensitivity of BBS. One probable reason might be that BBS assesses volitional balance control and does not account for or measure impairments in reactive balance. Secondly, it has shown to have a ceiling effect in healthy older adult population as it rates performance mostly during standing tasks (Newton, 1997; Langley and Mackintosh, 2007). Such tasks might not be challenging enough to assess dynamic balance control during daily living functional tasks in our population of community-dwelling, healthy older adults. Thus, the tasks performed and tested in BBS lack task-specificity to assess fall-resisting skills, thereby indicating its limited sensitivity for predicting fall-risk upon exposure to real-life like large external perturbations.

Psychosocial factors assessed in terms of balance confidence and fear of falling are crucial for fall prediction. However, balance confidence measured using ABC was not a significant fall-risk predictor. Previous studies done using ABC showed inconsistent results for ABC's ability to predict fall-risk (Lajoie and Gallagher, 2004; Schepens et al., 2010). While few studies demonstrated that ABC scores could differentiate fallers from non-fallers, with fallers having a lower ABC score (Mak and Pang, 2009; Hadjistavropoulos et al., 2011), other studies did not demonstrate a link between ABC scores and falls. The study results indicate the mean ABC score for our study population was 85.79%. However, despite having a high balance confidence score, suggesting a low risk of fall, over half of participants fell upon experiencing a slip or trip perturbation, indicating that ABC might demonstrate a ceiling effect as individuals may overestimate their balance abilities. Thus, there could be a potential mismatch between individuals' self-perception of their own balance abilities and their actual functional mobility, balance, and gait impairment, thus limiting ABC's sensitivity for fall prediction.

Our results indicated no correlation between 1-week steps data and commonly used clinical measurements for risk factors of falls, however a positive correlation was noted between 1-month steps data and BBS and ABC scores and a negative correlation with previous 1-year fall history. The correlation of step data with BBS could be expected considering the close association between mobility and stability and the BBS known to be a gold standard for assessing balance control in the older adults (Berg et al., 1992a; Santos et al., 2011). Previous studies have reported that enhancing one's mobility via isolated walking programs improves static and dynamic balance as well as overall postural stability (Brooke-Wavell et al., 1998; Paillard et al., 2004). Although there was a moderate positive correlation between these two variables, both variables were not selected as fall-risk predictors as discussed above.

The correlation between the ABC scale could also be expected and justified. It is known that any improvements in balance and stability may aid in reducing both fall-risk and the subsequent fear of falling in older adults (Gusi et al., 2012). Previous studies suggested that increased walking is associated with good balance perception (Yang and Hsu, 2010). As the ABC scale determines a person's own perception of balance activities, those who were more ambulatory and had more steps could have had an enhanced balance perception of themselves and vice versa. This might explain the paralleled finding of the positive relationship between steps and balance confidence. However, with the correlation between steps data and BBS and ABC being very modest (*r* < 0.3), the potential of smartphones to be used as a screening tool to identify older adults with reduced balance and balance confidence needs further investigation.

Additionally, the results of the sub-analysis found that long-term step monitoring yielded better association with commonly used clinical measures. Several studies have utilized and suggested that short-term monitoring for 1 week is adequate for PA monitoring and fall-risk (Tudor-Locke et al., 2005; Huberty et al., 2015). For example, a study done using wearable sensors in middle-aged and older women indicated that 24 h monitoring over a span of 1 week is a feasible approach for monitoring activity behavior (Huberty et al., 2015). However, there are several studies indicating that collecting long-term baseline data might be more accurate in yielding stochastic predictions (Mathie et al., 2004; Yang and Hsu, 2010). For example, a review article on PA monitoring suggested that long term monitoring could enable better understanding of PA behavior (Taraldsen et al., 2012). It is postulated that long-term data collection enables monitoring of day to day variability thereby providing a better understanding of consistent and habitual steps data of older adults and could thus show a better correlation with clinical fall-risk measures. While 1-week monitoring might be more feasible and could increase compliance, our results similar to few other studies suggest that 1-month monitoring might yield better results.

## Study Limitations

This study has several limitations. In the current study, the results could have been affected based on the hours and ways of phone carriage by participants as no strict instruction was given regarding phone usage. Further the software applications inbuilt or installed on the phone varied (e.g., iPhone vs. Android) which could have affected the step data accuracy. However, these factors could not be controlled due to the design of the study aiming at maximally collecting data in a natural manner and environment. Future studies could conduct studies with a uniform type of hardware (smartphone) and software (application) and additionally use wearable motion sensors to validate the association between daily steps data and older adults' fall-risk in response to laboratory-induced, real-life like external environmental perturbations. Lastly, study participants were among the healthiest community-dwelling older adults with a good physical performance and scores on clinical measurements. Hence, it is unknown whether current findings would also apply to frail older adults who are more susceptible to falls.

## Conclusion

The study revealed no association between smartphone steps data and laboratory fall-risk in a group of community-dwelling older adults with good physical performance. However, being the first of its kind, the current results could be leveraged to design further studies intending to use smartphone step data for fall-risk prediction. Further, the study reinforced previous findings that, older participants with fall histories and higher TUG scores were more likely to fall in the laboratory.

## Data Availability Statement

The raw data supporting the conclusions of this article will be made available by the authors, without undue reservation.

## Ethics Statement

The studies involving human participants were reviewed and approved by Institutional Review Board at the University of Illinois at Chicago. The patients/participants provided their written informed consent to participate in this study.

## Author Contributions

YW, RG, and TB made substantial contributions toward conception, design, and execution of the study. YW, RG, LK, and EW organized the data and performed statistical analysis. YW, RG, TB, EW, LK, and AS contributed in drafting the manuscript and revising it to create a final version for submission. All authors contributed to the article and approved the submitted version.

## Conflict of Interest

The authors declare that the research was conducted in the absence of any commercial or financial relationships that could be construed as a potential conflict of interest.
